# Association between haemoglobin A_1c_ and whole‐body heat loss during exercise‐heat stress in physically active men with type 2 diabetes

**DOI:** 10.1113/EP090915

**Published:** 2023-02-01

**Authors:** Nathalie V. Kirby, Robert D. Meade, Martin P. Poirier, Ronald J. Sigal, Pierre Boulay, Glen P. Kenny

**Affiliations:** ^1^ Human and Environmental Physiology Research Unit School of Human Kinetics University of Ottawa Ottawa Ontario Canada; ^2^ Harvard T.H. Chan School of Public Health Harvard University Boston MA USA; ^3^ Human Performance Research and Development Canadian Forces Morale and Welfare Services Ottawa Ontario Canada; ^4^ Departments of Medicine Cardiac Sciences, and Community Health Sciences Cumming School of Medicine Faculties of Medicine and Kinesiology University of Calgary Calgary Alberta Canada; ^5^ Clinical Epidemiology Program Ottawa Hospital Research Institute Ottawa Ontario Canada; ^6^ Faculty of Physical Activity Sciences University of Sherbrooke Sherbrooke Quebec Canada

**Keywords:** chronic disease, extreme heat, glycaemic control, haemoglobin A1c, physical activity

## Abstract

Type 2 diabetes is associated with a reduced capacity to dissipate heat. It is unknown whether this impairment is related to glycaemic control (indexed by glycated haemoglobin; haemoglobin A_1c_) is unknown. We evaluated the association between haemoglobin A_1c_ and whole‐body heat loss (via direct calorimetry), body core temperature, and heart rate in 26 physically active men with type 2 diabetes (43–73 years; HbA_1c_ 5.1–9.1%) during exercise at increasing rates of metabolic heat production (∼150, 200, 250 W m^−2^) in the heat (40°C, ∼17% relative humidity). Haemoglobin A_1c_ was not associated with whole‐body heat loss (*P* = 0.617), nor the increase in core temperature from pre‐exercise (*P* = 0.347). However, absolute core temperature and heart rate were elevated ∼0.2°C (*P* = 0.014) and ∼6 beats min^−1^ (*P* = 0.049), respectively, with every percentage point increase in haemoglobin A_1c_. Thus, while haemoglobin A_1c_ does not appear to modify diabetes‐related reductions in capacity for heat dissipation, it may still have important implications for physiological strain during exercise‐heat stress.

## Abstract (highlights)

- **What is the central question of this study?**
  Is the impairment in heat dissipation during exercise observed in men with type 2 diabetes related to glycaemic control (indexed by glycated haemoglobin; haemoglobin A_1c_)?
- **What is the main finding and its importance?**
  No association was found between haemoglobin A_1c_ (range: 5.1–9.1%) and whole‐body heat loss in men with type 2 diabetes during exercise in the heat. However, individuals with elevated haemoglobin A_1c_ exhibited higher body core temperature and heart rate responses. Thus, while haemoglobin A_1c_ is not associated with heat loss per se, it may still have important implications for physiological strain during exercise.

## 1 INTRODUCTION

Ageing is associated with a progressive decline in capacity to dissipate heat during exercise‐heat stress (D'Souza et al., 2020). This age‐related impairment is exacerbated in individuals with type 2 diabetes, resulting in greater body heat storage, core temperature, and heart rate during exercise relative to individuals without diabetes (Notley, Poirier et al., 2019). This may contribute to the greater risk of heat‐related injury or death in older individuals with type 2 diabetes (Kenny et al., 2016; Meade et al., 2020). However, factors influencing diabetes‐mediated reductions in heat dissipation are not fully understood.

Individuals with type 2 diabetes present with a range of glycaemic control, indexed by the proportion of glycated haemoglobin (HbA_1c_; normative range: 4–6%). HbA_1c_ ≤ 7% is considered good diabetes control (American Diabetes Association, 2021) and HbA_1c_ above this threshold is well‐known to increase the risk of diabetes‐related impairments (Steinberg et al., 1989), including reduced sweat gland innervation (Luo et al., 2012) and vascular endothelial function (Tai et al., 2021). These complications are thought to contribute to impaired heat loss responses of skin blood flow and sweating (Kenny et al., 2016), which facilitate whole‐body heat loss during exercise in the heat. While these associations suggest a potential link between glycaemic control and physiological capacity for heat dissipation, the association between HbA_1c_ and whole‐body heat loss during exercise in the heat has not been examined.

We therefore examined associations between HbA_1c_ and whole‐body heat loss, core temperature and heart rate responses during exercise in the heat in men with type 2 diabetes and a range of HbA_1c_ levels. We hypothesised that higher HbA_1c_ (indicating poorer glycaemic control) would be associated with impaired whole‐body heat loss. We also postulated that higher HbA_1c_ would also be associated with higher core temperature and heart rate responses to exercise in the heat as a result of reduced heat dissipation.

## 2 METHODS

This study was approved by the University of Ottawa Health Sciences and Science Research Ethics Board (H‐11‐20‐6234) and conformed to the *Declaration of Helsinki*, except for registration in a database. All participants provided written and informed consent.

### 2.1 Participants

Twenty‐six physically active (≥150 min per week of self‐reported physical activity) men aged 43–73 years who had been diagnosed with type 2 diabetes for ≥5 years, had no diabetes‐related complications, and were non‐smokers participated in this study (Table 1). Participants had a mean HbA_1c_ of 7.1% (range: 5.1–9.1%; 32–76 mmol mol^−1^). Some participants’ data (*n* = 17) have been reported previously as part of a study evaluating the effects of type 2 diabetes and heat acclimation on whole‐body heat loss (Notley, Poirier et al., 2019).

**TABLE 1:** Physical and diabetes‐related characteristics of middle aged to older men with uncomplicated type 2 diabetes (*n* = 26).

Characteristic	Mean (SD)	Range (min–max)
Age (years)	59 (7)	43–73
Height (m)	1.75 (0.05)	1.65–1.87
Mass (kg)	84.8 (13.5)	66.3–114.4
Body mass index (kg m^−2^)	27.7 (3.7)	20.9–36.2
Body surface area (m^2^)	2.00 (0.16)	1.71–2.11
${\dot{V}}_{O_{2}\mathrm{peak}}$ (ml kg^−1^ min^−1^)	32.3 (7.6)	19.7–50.8
Duration of type 2 diabetes (years)	11 (6)	5–26
HbA_1c_ (%)	7.1 (1.1)	5.1–9.1

Abbreviations: HbA_1c_, glycated haemoglobin; ${\dot{V}}_{O_{2}\mathrm{peak}}$, peak aerobic capacity.

### 2.2 Experimental design

Participants completed one screening session and one exercise heat‐stress test. Participants were instructed to avoid alcohol consumption and strenuous exercise for 24 h preceding all sessions. During the screening visit, height, body mass, body mass index, body surface area, peak oxygen uptake (${\dot{V}}_{O_{2}\mathrm{peak}}$; assessed via an incremental semi‐recumbent cycling exercise protocol) and self‐reported physical activity levels (Tremblay et al., 2011) were assessed.

### 2.3 Exercise‐heat stress test

Upon arrival at the laboratory, participants provided a urine sample to confirm euhydration (urine specific gravity ≤1.025) and a nude body mass was obtained. Participants then donned athletic shorts and sandals and were instruments in a thermoneutral room (∼24°C). They then entered the direct air calorimeter (40°C, ∼17% relative humidity). Following 30 min seated rest, participants completed three 30 min bouts of semi‐recumbent cycling at increasing fixed rates of metabolic heat production of 151 (12) W m^−2^ (light; ∼40%${\dot{V}}_{O_{2}\mathrm{peak}}$), 202 (16) W m^−2^ (moderate; ∼50%${\dot{V}}_{O_{2}\mathrm{peak}}$) and 254 (14) W m^−2^ (vigorous; ∼65%${\dot{V}}_{O_{2}\mathrm{peak}}$), each followed by 15‐min rest. Participants were not permitted to drink during the protocol. This progressive heat stress protocol has been used to evaluate the influence of numerous individual factors on the physiological capacity for heat dissipation (e.g., age, sex, hydration; D'Souza et al., 2020; Gagnon & Kenny, 2012; Meade, Notley, D'Souza et al., 2019) using an incremental exercise protocol in a manner similar to the evaluation of ${\dot{V}}_{O_{2}\mathrm{peak}}$ (Meade, Notley, & Kenny, 2019). This protocol employs a fixed rate of heat production to maintain a similar thermal drive for evaporative heat loss amongst participants (Kenny & Jay, 2013). We did not impose restrictions on the time of year of testing (spring: *n* = 15; summer: *n* = 2; autumn: *n* = 3; winter: *n* = 6).

### 2.4 Measurements

The modified Snellen direct air calorimeter was used to obtain a continuous measure of whole‐body heat loss (dry + evaporative heat loss) (Kenny & Jay, 2013). Evaporative heat loss was calculated as the calorimeter outflow–inflow difference in absolute humidity (measured with high‐precision dew point hygrometers; model 373‐H, RH Systems, Albuquerque, NM, USA) multiplied by the air mass flow and latent heat of vaporization of sweat (2426 J g^−1^). Dry heat loss was calculated using the outflow–inflow air temperature difference (measured with high‐precision temperature sensors (± 0.002°C); Black Stack model 1560; Hart Scientific, American Fork, UT, USA) and specific heat capacity of air (1005 J kg^−1^°C^−1^). Note that dry heat loss was measured as a negative value (i.e., heat gain), since ambient temperature was higher than skin temperature. Metabolic rate was measured using an automated indirect calorimetry system (Moxus modular metabolic system; AEI Technologies, Bastrop, TX, USA). Metabolic heat production was calculated as metabolic rate minus the rate of external work (Kenny & Jay, 2013).

Core temperature was measured continuously as either rectal (*n* = 19), oesophagal (*n* = 6), or gastro‐intestinal temperature (*n* = 1). Rectal and oesophageal temperatures were measured using a thermocouple probe (Mon‐a‐therm General Purpose Temperature Probe, Mallinckrodt Medical, St. Louis, MO, USA) inserted 12 cm past the anal sphincter or 40 cm past the nostril, respectively. Gastro‐intestinal temperature was measured via a telemetric pill (VitalSense ingestible capsule thermometer; Mini Mitter Company, Bend, OR, USA). Heart rate was recorded using a Polar H10 monitor (Polar Electro Oy, Kempele, Finland).

### 2.5 Data and statistical analysis

Statistical analyses to evaluate the associations between HbA_1c_ and whole‐body heat loss and its derivatives (i.e., evaporative and dry heat loss), core temperature and heart rate were performed using an average of the final 5 min of each exercise bout. Three participants did not complete the final exercise bout due to volitional fatigue. Thus, the values reported for the vigorous‐intensity exercise bout represent *n* = 23 participants.

**FIGURE 1:**
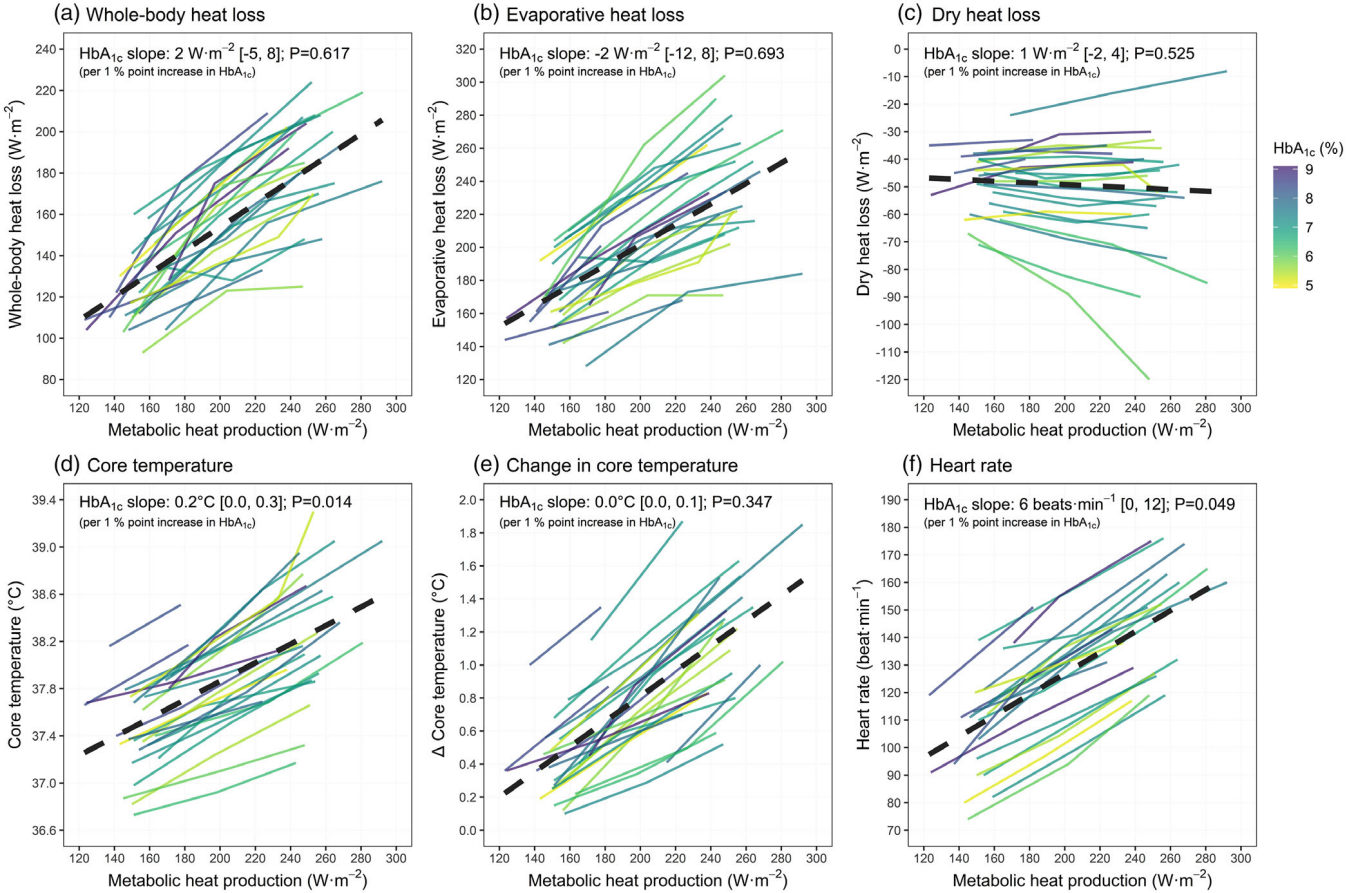
Associations of metabolic heat production and glycated haemoglobin (HbA1_c_) with whole‐body (evaporative + dry) heat loss (a), evaporative heat loss (b), dry heat loss (c), core temperature (d), change in core temperature from pre‐exercise (e), and heart rate (f) in men with type 2 diabetes during exercise in the heat (40°C, ∼17% relative humidity). Dashed lines represent the mean slope across all levels of metabolic heat production, and continuous lines represent individual participant data, coloured according to percentage of HbA_1c_. All outcomes include data from *n* = 26 participants, except heart rate, which includes data from *n* = 24 participants. The HbA_1c_ slope refers to the estimated mean [95% confidence interval] change in each outcome variable per percentage point increase in HbA_1c_ at a given heat production. *P* < 0.050 was considered statistically significant.

Data were analysed using linear mixed effects models. Fixed and random effects and variance/covariance structures were determined using Akaike's information criterion. The fixed effects included metabolic heat production and HbA_1c_. For all analyses, an additive model (i.e., a model *without* a heat production × HbA_1c_ interaction) provided a better fit (lower Akaike's information criterion value) than a multiplicative model (i.e., a model *with* a heat production × HbA_1c_ interaction; all interaction terms *P* = 0.067 ‐ 0.946) . As such, reported models assume that the effect of HbA_1c_ on each outcome variable is independent of the level of heat production. The coefficient/slope for HbA_1c_ reported in the results in Figure 1, therefore, reflect the magnitude by which a 1 percentage point increase in HbA_1c_ increases the outcome variable at any given heat production. Random effects were included to account for repeated measures since we incorporated data from all exercise bouts into a single model for each outcome. For the random effects structure, we modelled either a random intercept (participant ID) or random intercept and slope (participant ID × heat production).

Aerobic fitness is associated with HbA_1c_ (Larose et al., 2011) and whole‐body heat loss capacity (Notley, Lamarche et al., 2019). In sensitivity analyses we therefore evaluated whether including ${\dot{V}}_{O_{2}\mathrm{peak}}$ as a fixed effect in the models described above had an effect on the relations between HbA_1c_ and the study outcomes. As a simple test to assess the potential for confounding by other participant characteristics, we also evaluated Pearson correlations between participant characteristics (age, height, body mass, body surface area, body mass index, and duration of type 2 diabetes) and both HbA_1c_ and whole‐body heat loss.

For all analyses, a two‐sided *P* < 0.050 was considered statistically significant. Homoscedasticity and normality of residuals were evaluated by visual assessment of residuals and Q–Q plots. Descriptive statistics are presented as means (standard deviation) and the slopes of the relations between HbA_1c_ and the study outcomes as mean (95% confidence limits). R statistical software (Version 4.2.0, R Core Team, R Foundation for Statistical Computing, Vienna, Austria) was used for all analyses and data visualizations.

## 3 RESULTS

Whole‐body heat loss (Figure 1a) and evaporative heat loss (Figure 1b) were elevated with increasing metabolic heat production (both *P* < 0.001). By contrast, dry heat loss (Figure 1c) did not differ as a function of heat production (*P* = 0.250). Neither whole‐body heat loss (*P* = 0.617), evaporative heat loss (*P* = 0.693), nor dry heat loss (*P* = 0.525) were associated with HbA_1c_ (Figure 1a–c). Core temperature (Figure 1d) increased with heat production (*P* < 0.001) and, for a given heat production, was elevated 0.2°C (95% CI: 0.0, 0.3) with each percentage point increase in HbA_1c_ (*P* = 0.014). However, no association with HbA_1c_ was observed when core temperature was expressed as a change from pre‐exercise (*P* = 0.347; Figure 1e). Like absolute core temperature, heart rate rose with heat production (*P* < 0.001) and was 6 (95% CI: 0, 12) beats min^−1^ higher for each percentage point rise in HbA_1c_ (*P* = 0.049; Figure 1f).

Modelling ${\dot{V}}_{O_{2}\mathrm{peak}}$as a fixed effect in sensitivity analyses did not alter findings for the associations between HbA_1c_ and whole‐body heat loss, evaporative heat loss, or dry heat loss (all *P* ≥ 0.330). Likewise, including ${\dot{V}}_{O_{2}\mathrm{peak}}$ in the model did not substantially alter the slope of the relation between HbA_1c_ and core temperature, whether presented as absolute values (HbA_1c_ slope: 0.2°C (95% CI: 0.0, 0.3), *P* = 0.009) or as a change from pre‐exercise (*P* = 0.198). While the association of HbA_1c_ with heart rate was also similar to the primary model (HbA_1c_ slope: 6 (95% CI: 0, 13) beats min^−1^), the slope coefficient was no longer statistically significant (*P* = 0.062). Consistent with these findings, we did not detect a significant correlation between ${\dot{V}}_{O_{2}\mathrm{peak}}$ and HbA_1c_ (*r* = −0.24, *P* = 0.232), though there was a weak but significant correlation between ${\dot{V}}_{O_{2}\mathrm{peak}}$ and whole‐body heat loss (*r* = 0.27, *P* = 0.018). Similarly, correlations between participant characteristics (age, height, body mass, body surface area, body mass index and type 2 diabetes duration) and both HbA_1c_ (*r*: −0.14 to 0.35, all *P* ≥ 0.088) and whole‐body heat loss (*r* = −0.15 to 0.06, all *P* ≥ 0.128) were not statistically significant.

## 4 DISCUSSION

We examined whether glycaemic control, as quantified by HbA_1c_, influenced whole‐body heat loss, core temperature, and heart rate during exercise in the heat in habitually active men with type 2 diabetes. Contrary to our hypothesis, HbA_1c_ was not associated with whole‐body heat loss. Consequently, the increase in core temperature from pre‐exercise was unaffected by HbA_1c_. However, at any given rate of metabolic heat production, absolute core temperature was elevated by ∼0.2°C and heart rate by ∼6 beats min^−1^ with every percentage point increase in HbA_1c_, effects which were largely independent of aerobic fitness. These data demonstrate that, while HbA_1c_ does not appear to modify diabetes‐related reductions in the capacity for heat dissipation previously observed by our laboratory (Notley, Poirier et al., 2019), individuals with poor glycaemic control may still experience greater thermal and cardiovascular strain during exercise in the heat.

Given similarities in whole‐body heat loss and rises in core temperature across levels of HbA_1c_, the observed association between HbA_1c_ and absolute core temperature implies that basal core temperature increases with higher HbA_1c_. Indeed, in *post hoc* analyses, we observed a moderate, though non‐statistically significant, correlation between HbA_1c_ and pre‐exercise core temperature (*R* = 0.31, *P* = 0.13). This is consistent with data from Gubin et al. (2017), who observed elevated 24‐h core temperature with worsening disease state (i.e., from the control group to pre‐diabetes to diagnosed type 2 diabetes of ≥5 years). Notably, heat‐related injury occurrence coincides with absolute temperature thresholds; heat stroke, for example, occurs most commonly at core temperatures >40°C (Kenny et al., 2018). Elevated core temperatures with worsening glycaemic control may therefore contribute to the increased risk of adverse health events in individuals with type 2 diabetes (Kenny et al., 2016; Meade et al., 2020), though future work is needed to confirm this.

Regular exercise is recommended for diabetes management, as physical activity increases insulin sensitivity and thus is critical for improving blood glucose control (Kanaley et al., 2022). However, as the planet experiences more frequent and enduring temperature extremes as well as hotter average summer temperatures, it becomes challenging to manage the disease with regular exercise while heading health guidelines to avoid exercise in hot weather (Kenny et al., 2016). To combat this, practical heat‐health strategies such as reducing exercise intensity on hot days or limiting exercise to the early morning or evening when outdoor temperatures are lower are needed. These strategies are especially pertinent to consider for individuals with poor glycaemic control, who may experience an exacerbated risk of reaching potentially dangerous end‐exercise core temperatures superimposed on an already impaired capacity for heat loss associated with type 2 diabetes (Notley, Poirier et al., 2019).

### 4.1 Limitations

Limitations of this study include enrolling a male‐only cohort and that participants were all physically active (≥150 min per week), meaning that they likely do not represent the most heat‐vulnerable among those with type 2 diabetes (Kenny et al., 2016). Further, while our model selection criteria suggested an additive relation between HbA_1c_ and our study outcomes, we do not discount the possibility that, in some circumstances (e.g., sedentary individuals), the association between higher HbA_1c_ and thermoregulatory (heat loss and core temperature) and/or cardiovascular responses could be exacerbated with increasing heat production, since other individual factors affecting heat loss are heat load‐dependent (e.g., sex and age; D'Souza et al., 2020; Gagnon & Kenny, 2012). However, this effect, if any, is likely small. Relatedly, while we enrolled a larger sample size than is typical in environmental physiological studies (Twomey et al., 2021), we were unable to evaluate the influence of multiple potential confounders or modifiers of the relation between HbA_1c_ and thermoregulatory function simultaneously. Finally, we did not enrol participants with HbA_1c_ > 9.1%. It is possible that individuals with higher HbA_1c_ levels might have had greater impairments in capacity for heat loss.

### 4.2 Conclusions

We did not observe an association between HbA_1c_ and whole‐body heat loss in physically active men with type 2 diabetes exercising in the heat. However, we did detect alterations in body core temperature and heart rate with increasing HbA_1c_, which were largely independent of aerobic fitness. While these findings suggest that higher HbA_1c_ levels may be associated with greater susceptibility to potentially dangerous elevations in core temperature and cardiovascular strain, additional work is required to evaluate this hypothesis.

## AUTHOR CONTRIBUTIONS

Nathalie V. Kirby and Glen P. Kenny conceptualized and designed the research; Nathalie V. Kirby and Martin P. Poirier performed data collection; Nathalie V. Kirby and Robert D. Meade performed statistical analyses, interpreted results, and created the data visualisations; Nathalie V. Kirby and Robert D. Meade drafted the manuscript; all authors revised the manuscript. All authors have read and approved the final version of this manuscript and agree to be accountable for all aspects of the work in ensuring that questions related to the accuracy or integrity of any part of the work are appropriately investigated and resolved. All persons designated as authors qualify for authorship, and all those who qualify for authorship are listed.

## CONFLICT OF INTEREST

None.

## Supporting information

Statistical Summary Document

Raw data

## Data Availability

Raw data are included under Supporting Information.
